# Supplementary material for: Determination of Cannabinoids in Meat Products and Animal Feeds in Singapore Using Liquid Chromatography–Tandem Mass Spectrometry
Source: Foods. 2024 Aug 18;13(16):2581. doi: 10.3390/foods13162581 (PMC11353810; doi:10.3390/foods13162581)
Supplement: Supplementary file 1 [file foods-13-02581-s001.zip › foods-3112560-supplementary.pdf]

foods

Article

Determination of cannabinoids in meat products and animal feeds in Singapore using liquid chromatography-tandem mass spectrometry

Jia En Valerie Sin[1], Ping Shen[1*], Lifei Huang[1], Yuansheng Wu[1], Sheot Harn Chan[1,2]

1. National Centre for Food Science, Singapore Food Agency, 7 International Business Park, Singapore 609919, Singapore
2. Department of Food Science & Technology, National University of Singapore, 2 Science Drive, Singapore 117543, Singapore

* Correspondence: author

Abstract: There has been a growing interest in the use of hemp as an animal feed ingredient considering its economic value and nutritional properties. However, there is a paucity of research regarding the safety of hemp-based animal feed currently. Thus, this raises safety concerns on the potential transfer of cannabinoids from hemp-based animal feed to animal products intended for human consumption and its health effects. As such, the detection and quantification of cannabinoids in meat and animal feeds would be desirable for monitoring purposes. In this study, a simple, rapid and sensitive method for the simultaneous quantification of 4 major cannabinoids in meat and animal feeds by liquid chromatography-tandem mass spectrometry (LC-MS/MS) was successfully developed and validated. The method was selective and sensitive, achieving limits of detection and quantification for the 4 cannabinoids from 5 to 7 μg/kg and 15 to 21 μg/kg respectively. The overall recovery with matrix match calibration curves for the cannabinoids ranged from 87-115%. The coefficients of variation were between 2.17-13.38% for intraday precision and 3.67-12.14% for inter-day precision. The method was subsequently applied to monitor cannabinoids in 120 meat and 24 animal feed samples. No cannabinoid was detected, suggesting no imminent food safety concerns arising from the potential incorporation of hemp and by-products in animal feed and nutrition under the promotion of sustainable agricultural practices.

Keywords: Cannabinoid, hemp, liquid chromatography-tandem mass spectrometry (LC-MS/MS), feed, animal tissue

foods

MDPI

Supplementary Materials

Table S1. Signal intensities of target analytes for spiked samples processed using 0.2 µm nylon membrane filter versus Oasis PRiME HLB SPE cartridge at 5µg/kg and 10 µg/kg

Target Analyte	5 µg/kg		10 µg/kg	
	0.2 µm nylon membrane filter (E+04)	Oasis PRiME HLB SPE cartridge (E+04)	0.2 µm nylon membrane filter (E+04)	Oasis PRiME HLB SPE cartridge (E+04)
**Delta-9-tetrahydrocannabinol (THC)**	**2.40**	**2.80**	**6.20**	**7.00**
**Cannabidiol (CBD)**	**2.30**	**3.30**	**6.50**	**6.60**
**Cannabinol (CBN)**	**2.30**	**2.00**	**6.70**	**4.40**
**Tetrahydrocannabinolic acid (THCA)**	**8.50**	**1.00**	**19.0**	**4.00**

Table S2. Chemical structures and MS/MS spectra of product ions

<table>
<tr><th>Analyte</th><th>Precursor ion (m/z)</th><th>Product ion (m/z)</th><th>MS/MS spectrum</th></tr>
<tr><td>Delta-9-tetrahydrocannabinol (THC)</td><td>315.2</td><td>193.2
259.2
123.1</td><td>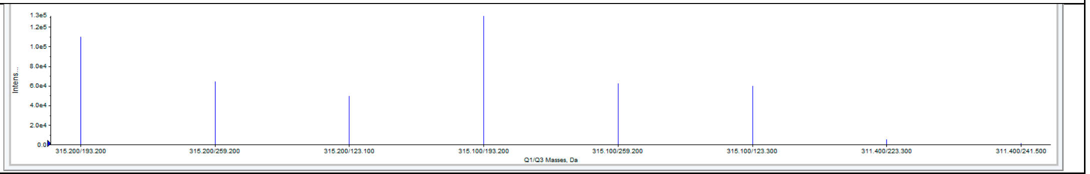
</td></tr>
<tr><td>Cannabidiol (CBD)</td><td>315.1</td><td>193.2
259.2
123.3</td><td>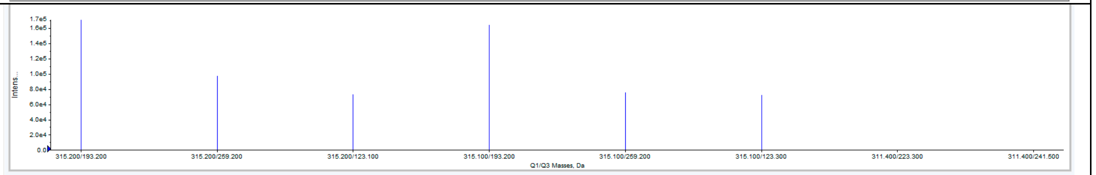
</td></tr>
<tr><td>Cannabinol (CBN)</td><td>311.4</td><td>223.3
241.5</td><td>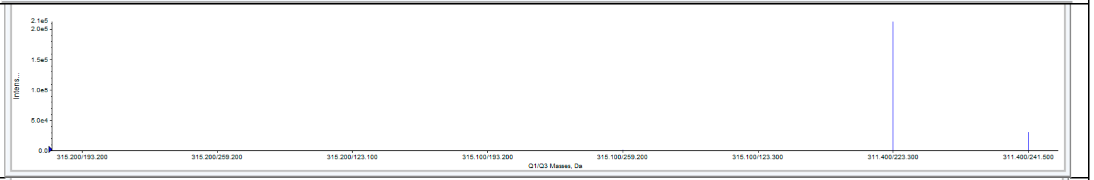
</td></tr>
<tr><td>Tetrahydrocannabinolic acid (THCA)</td><td>357.0</td><td>191.2
245.0
179.0</td><td>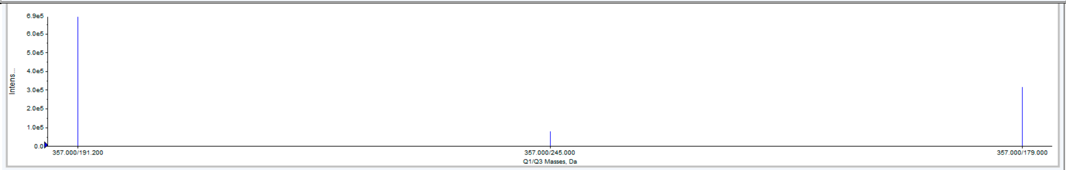
</td></tr>
</table>
